# Autologous extracellular Hsp70 exerts a dual role in rheumatoid arthritis

**DOI:** 10.1007/s12192-020-01114-z

**Published:** 2020-05-01

**Authors:** Stefan Tukaj, Jagoda Mantej, Michał Sobala, Katarzyna Potrykus, Krzysztof Sitko

**Affiliations:** 1 Department of Molecular Biology, Faculty of Biology, University of Gdańsk, Wita Stwosza 59, 80-308 Gdańsk, Poland; 2 Department of Bacterial Molecular Genetics, Faculty of Biology, University of Gdańsk, Gdańsk, Poland

**Keywords:** Heat shock proteins, HSP, T helper cell (Th) populations, Rheumatoid arthritis, RA, Hsp70

## Abstract

Extracellular heat shock proteins (Hsp) influence the adaptive immune response and may ameliorate pathogenesis of autoimmune diseases. While some preclinical observations suggest that highly conserved bacterial and/or murine Hsp70 peptides have potential utility in treatment of rheumatoid arthritis (RA) via induction of T regulatory cells (Treg), the role of extracellular inducible human Hsp70 in adaptive immune processes requires further investigation. The present study evaluated Hsp70 influence on inflammatory cytokine-mediated modulation of T cell immunophenotype in ways that influence RA onset and severity. Initial experiments in the present investigation revealed that serum levels of Hsp70 are approximately 2-fold higher in RA patients versus healthy control subjects. To explore the effect of extracellular Hsp70 on key processes underlying the adaptive immune system, the effects of a highly pure, substrate-, and endotoxin-free human Hsp70 on polarization of the T helper cell subpopulations, including CD4^+^IL-17^+^ (Th17), CD4^+^FoxP3^+^ (Treg), CD4^+^IFN-γ^+^ (Th1), and CD4^+^IL-4^+^ (Th2), were studied in naïve human peripheral blood mononuclear cell (PBMC) cultures stimulated with anti-CD3/28 mAb. Major findings included an observation that while Hsp70 treatment increased Th17 frequencies and Th17/Treg ratio, the frequency of Th1 cells and the Th1/Th2 ratio were significantly decreased in the Hsp70-treated PBMC cultures. Moreover, data shown here provides preliminary suggestion that major contributing Hsp70-mediated immunomodulation includes interleukin 6 (IL-6) influence on Th17/Treg and Th1/Th2, since expression of this inflammatory cytokine is enhanced by in vitro Hsp70 treatment. These results are nevertheless preliminary and require further investigation to validate the above model.

## Introduction

Rheumatoid arthritis (RA) is one of the most common chronic autoimmune diseases characterized by synovial inflammation and bone erosion. This disease affects up to 1% of the worldwide population. The pathogenesis of RA is driven by an inflammatory network in which both the innate and adaptive immune systems, including the immune complex-mediated complement activation, and the pro-inflammatory cytokine networks support the disease progression (Firestein and McInnes 2017). Cells of the adaptive immune systems play a critical role in autoimmunity. Dysregulation of this arm of the immune response seems to be responsible for the development of RA (Angelotti et al. 2017) and is influenced by both the genetic and environmental factors. In the past, RA was considered as an autoimmune disease driven by pro-inflammatory CD4^+^IFN-γ^+^ (Th1) T helper cells which predominate over the CD4^+^IL-4^+^ (Th2) subset that exerts regulatory functions (Schulze-Koops and Kalden 2001). Nowadays, both the Th1 and the pro-inflammatory CD4^+^IL-17^+^ (Th17) T helper cells are thought to play an important role in the initiation of RA (McInnes and Schett 2011). The balance between Th17 and T regulatory (Treg) cells is crucial for the immune homeostasis protecting against the autoimmune response in RA (Schinnerling et al. 2017).

Cell-protecting heat shock proteins (Hsp) possess a dual role in the immune-mediated disorders, i.e., they are involved in the induction and propagation of autoimmune diseases, but also play a role in suppressing them. Such an equivocal role of those chaperones depends on the Hsp class, the site of inflammation, and the type of disease (Radons 2016; Pockley and Henderson 2018). Increased levels of Hsp70 and autoantibodies to Hsp70 in sera of RA or juvenile idiopathic arthritis (JIA) patients are found to be associated with the disease progression and activity (Zlacka et al. 2006; Najafizadeh et al. 2015), and numerous in vitro studies indicated pro-inflammatory properties for Hsp70 in their interactions with the innate immune cells. However, some of these later inflammatory effects may have resulted from the presence of endotoxins in the recombinant protein preparations (Borges et al. 2012; Pockley and Henderson 2018). In contrast to the reported Hsp70 pro-inflammatory properties, a body of literature indicates that these chaperones can have profound anti-inflammatory effects and the presence of correlations between the disease activity and Hsp70 expression may be an accompanying phenomenon (van Eden 2018). Pre-clinical observations have proven that artificial immunization with highly conserved bacterial and/or murine Hsp70 peptides could be regarded as a potential treatment target for rheumatoid arthritis (RA) via induction of antigen-specific Treg cells (Wendling et al. 2000; van Eden et al. 2005; Wieten et al. 2009; van Herwijnen et al. 2012; van Eden et al. 2013; Tukaj and Kaminski 2019). Nevertheless, the role of extracellular inducible human Hsp70 in adaptive immune processes requires further investigation.

Here, the effects of highly pure, substrate-, and endotoxin-free human extracellular Hsp70 on polarization of the major human T helper cell subsets, such as Th1, Th2, Th17, and Treg, are explored in vitro in the context of RA development.

## Materials and methods

### Patients and controls

Twenty-seven patients with RA, fulfilling the ACR classification criteria from 1987 for this disease, and 37 healthy controls were included in this study. The disease activity was assessed according to the Disease Activity Score, including 28 joint counts (DAS 28-ESR = DAS 28), and joint damage was evaluated based on the Steinbrocker radiographic criteria. The use of human biological material was approved by the Ethics Committee of the Medical University of Gdańsk, Poland, and written informed consent was obtained according to the Declaration of Helsinki.

### Cloning, expression, and purification of human Hsp70

Synthetic DNA fragment encoding Hsp70 (HSPA1A) from *Homo sapiens* (NP_005336.3) has been obtained from Thermo Scientific (GeneArt service). Codon usage was optimized for efficient gene expression in *E. coli* by the GeneOptimizer software. The insert was synthesized with N-terminal 6x-His-SUMO tag and cloned into the pET151/TOPO (Thermo Scientific) plasmid. Lipopolysaccharide-free *E. coli* BL21 (DE3) ClearColi (Lucigen) strain carrying the plasmid was grown in the LB medium supplemented with 1% NaCl, 1 mM IPTG (Sigma), and ampicillin at 18 °C, overnight. The use of ClearColi cells warrants that the purified overproduced protein will be free of endotoxin and lipopolysaccharide contaminants. Cells were harvested by centrifugation, resuspended in a lysis buffer (20 mM Tris-HCl pH 8.0, 500 mM NaCl, 20 mM imidazole, 10% glycerol, ROCHE protease inhibitor cocktail), and disrupted by sonication. After centrifugation, the supernatant was loaded on the HIS-Select® Nickel Affinity Gel resin (Sigma) equilibrated with the lysis buffer. To remove unbound proteins and the chaperone-associated substrates, the column was washed with a buffer containing 5 mM ATP, 5 mM MgCl_2_, 1 M NaCl, and 20 mM Tris-HCl pH = 8.0. The Hsp70 containing fractions (eluted with lysis buffer containing 180 mM imidazole) were dialyzed against a dialysis buffer (20 mM Tris-HCl pH = 8.0, 250 mM NaCl, 10% glycerol), followed by His-tag cleavage using SUMO protease (Sigma). To remove His-tag from the mixture, the protein sample was loaded on the HIS-Select® Nickel Affinity Gel resin (Sigma) equilibrated with the dialysis buffer. The Hsp70 fraction (99% purity) was filtered (0.22 μm) and stored at − 80 °C.

### PBMC culture

Peripheral blood mononuclear cells (PBMCs) were isolated from the venous blood of healthy volunteers by Histopaque 1077 (Sigma) gradient centrifugation and cultured as described previously, with a minor modification (Tukaj et al. 2014). Briefly, PBMCs were washed with PBS and resuspended at 0.5 × 10^6^–1 × 10^6^ cells per ml of the medium (RPMI 1640 supplemented with 10% fetal calf serum and 1% penicillin/streptomycin; all components from Sigma). Cells were cultured in the presence of 1 μg/ml immobilized anti-CD3ε mAb (BioLegend) and 1 μg/ml soluble anti-CD28 mAb (BioLegend), in 24-well culture plates, at 5% CO_2_ and at 37 °C without or with different concentrations (1 or 10 μg/ml) of the human Hsp70 for 72 h.

### Detection of extracellular Hsp70

Serum Hsp70 levels were assessed by using a commercially available HSP70 High Sensitivity (Sandwich) ELISA kit (Abcam), according to the manufacturer’s instructions. The kit is designed to assay human Hsp70 in serum or plasma. Quantitative determination of HSP70 in the serum was performed based on a standard curve. The sensitivity or limit of detection of the assay is 90 pg/ml.

### Cytokine detection

Cell culture supernatant levels of human IL-6 were assessed by ELISA (BioLegend) prior the monensin/PMA/ionomicin addition, according to the manufacturer’s instructions. Quantitative determination of IL-6 was performed based on a standard curve.

### FACS immunophenotyping

Anti-CD3/28 mAb-stimulated human PBMCs were cultured with or without the human Hsp70 protein (1 or 10 μg/ml) for 72 h h. In the case of Th1/Th2/Th17 phenotyping, monensin (BioLegend), phorbol-12-myristate-13-acetate (PMA) (50 ng/ml; Sigma), and ionomycin (1 μg/ml; Sigma) were added for the last 4 h of culture. Cells were washed, stained for the T helper cells (anti-CD4-FITC; BioLegend), fixed, permeabilized, and stained for detection of intracellular molecules using the following anti-human mAbs: anti-IFN-γ-APC, anti-IL-17-APC, anti-FoxP3-APC, and anti-IL-4-PE (BioLegend). Samples were analyzed using the CyFlow Cube 6-flow cytometer (Sysmex).

### Statistical analysis

Statistical analyses were performed using the GraphPad Prism 5 (San Diego, CA) software. The Shapiro-Wilk test was used to verify whether the data had normal distribution. Non-normal distributed data was analyzed by the Mann Whitney *U* test, Kruskal-Wallis test, and Spearman’s rank correlation test. *P* values that were less than 0.05 were considered as statistically significant.

## Results

### Serum levels of Hsp70 are significantly increased in the rheumatoid arthritis patients

**Fig. 1:**
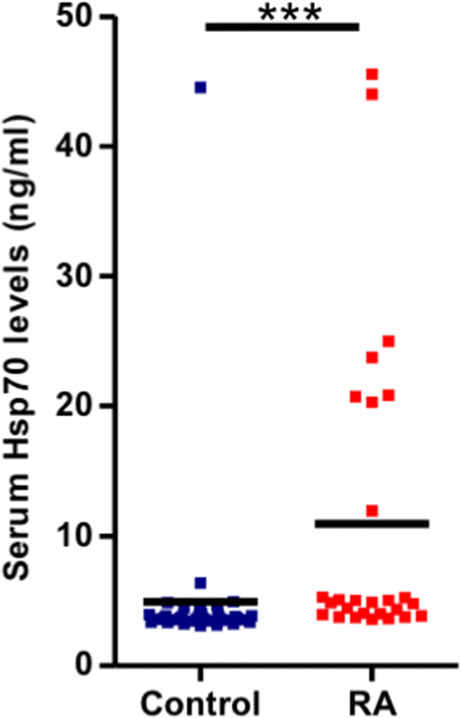
Levels of Hsp70 are increased in the sera of patients with rheumatoid arthritis (RA). Serum levels of Hsp70 in RA (*n* = 27) and age- and gender-matched healthy controls (*n* = 37) were assessed by an enzyme-linked immunosorbent assay. The squares and horizontal bars indicate individual and mean values in each group, respectively. ***P < 0.0001

We found that the serum levels of Hsp70 are significantly higher (*p* < 0.0001) in the RA patients (*n* = 27) when compared to the age- and gender-matched healthy controls (*n* = 37), as assessed by ELISA (Fig. 1). The levels of Hsp70, however, were neither associated with the disease activity (*p* = 0.85) nor the disease’s progression based on the radiological Steinbrocker RTG criteria (*p* = 0.2) in the RA patients.

### Extracellular Hsp70 induces interleukin 6 (IL-6) secretion, stimulates Th17, and inhibits Th1 frequencies in the human PBMC cultures

**Fig. 2:**
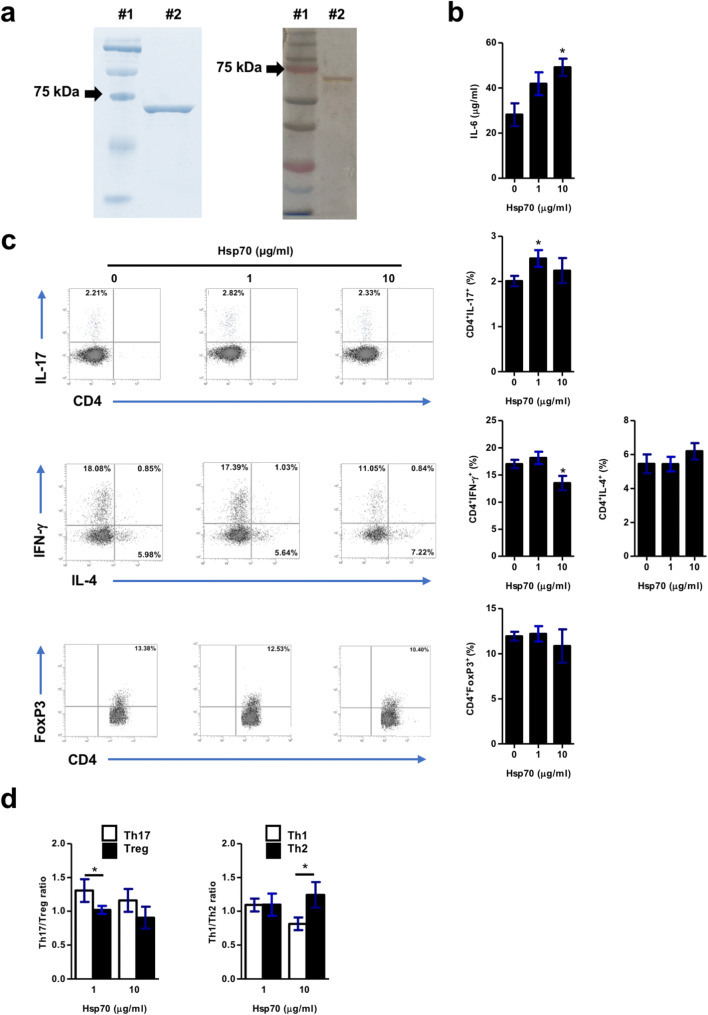
Hsp70 induces IL-6 secretion, stimulates Th17, and inhibits Th1 frequencies in vitro*.* **a** Affinity-purified human Hsp70 was resolved in 10% SDS-PAGE gel and stained with Coomassie Brilliant Blue (*left*) and silver staining (*right*). Line #1: molecular weight ladder; line #2: Hsp70. **b** IL-6 levels in supernatants from human PBMC cultures stimulated with anti-CD3/CD28 mAb antibody for 68 h in the absence or presence of Hsp70 (1 or 10 μg/ml). **c** Frequencies of Th17, Th1, Th2, and Treg cell populations in human PBMC cultures stimulated for 72 h with anti-CD3/CD28 mAb in the absence or presence of Hsp70 (1 or 10 μg/ml) were analyzed by flow cytometry. The numbers in the gates of the representative results are the percentages of the respective cell populations with respect to the total cell numbers. **d** The Th17/Treg and Th1/Th2 ratios in PBMC cultures stimulated with anti-CD3/CD28 mAb for 72 h in the presence of Hsp70 (1 or 10 μg/ml). To express the ratios, data were normalized to a mean value of the respective controls. The results are presented as mean values ± SEM of *n* = 8. The experiments were repeated twice. *P < 0.05

The effects of highly pure, substrate-, and endotoxin-free human Hsp70 (Fig. 2a) on secretion of IL-6 and polarization of the T helper cell populations [such as CD4^+^IL-17^+^ (Th17), CD4^+^IFN-γ^+^ (Th1), CD4^+^IL-4^+^ (Th2), and CD4^+^FoxP3^+^ (Treg)] were studied in the anti-CD3/28-stimulated naïve human peripheral blood mononuclear cell (PBMC) cultures. Hsp70 stimulated secretion of IL-6 in a dose-dependent manner (Fig. 2b). While Hsp70 induced the Th17 frequencies, the Th1 frequencies were significantly decreased in the Hsp70-treated PBMC cultures. No significant effects of Hsp70 on Treg and Th2 frequencies were found (Fig. 2c). In addition, Hsp70 induced the Th17/Treg ratio and decreased the Th1/Th2 ratio in the PBMC cultures in a dose-dependent fashion, i.e., lower concentrations of Hsp70 (1 μg/ml) induced the Th17/Treg ratio, and higher concentrations of Hsp70 (10 μg/ml) inhibited the Th1/Th2 ratio (Fig. 2d).

## Discussion

Numerous data have indicated that the heat shock proteins (Hsp) are overexpressed in inflamed tissues. Whether upregulation of Hsp in chronically inflamed tissues regulates or participates in the pathology process remains unclear. On one hand, blockade of the Hsp90 chaperone activity has led to inhibition of the (auto)immune response (Tukaj and Węgrzyn 2016). On the other hand, pharmacological induction of Hsp70 in cells has down-regulated the inflammation process in preclinical models of arthritis (Wieten et al. 2007, 2010a, b; Tukaj and Węgrzyn 2016; Tukaj and Kaminski 2019). Moreover, polyphenol-mediated induction of Hsp32 (heme oxygenase-1) resulted in dramatic improvements in the prognosis of osteoarthritis symptoms in the human population (Mahmoud et al. 2015).

Preclinical observations suggest that the highly conserved bacterial/murine extracellular Hsp70 could be regarded as a potential treatment target for RA (Wendling et al. 2000; van Eden et al. 2005; Wieten et al. 2009; van Herwijnen et al. 2012; van Eden et al. 2013; Tukaj and Kaminski 2019). Physiological role of its human counterpart in the adaptive arm of the immune system, however, requires further investigation. Here, we found that the serum levels of Hsp70 are significantly higher in the RA patients when compared to healthy controls. However, the serum levels of Hsp70 did not correlate with the disease activity and progression in RA patients. In this study, PBMC derived from healthy donors were used to explore the role of increased concentrations of Hsp70 in T helper cell polarization in the context of RA development. The Hsp70 used were highly pure, endotoxin-, and substrate-free. We found inhibitory effects of the extracellular Hsp70 on Th1 frequencies and the Th1/Th2 ratio in the anti-CD3/28-stimulated naïve PBMC cultures. This may suggest an immunosuppressive activity of the extracellular Hsp70 because RA is considered as an autoimmune disease driven by the pro-inflammatory Th1 cells. These cells predominate over the Th2 subset that exerts regulatory functions (Schulze-Koops and Kalden 2001). On the other hand, Hsp70 induced the Th17 frequencies and the Th17/Treg ratio in the investigated PBMC cultures, pointing towards its pro-inflammatory activity. The pro-inflammatory activity of extracellular Hsp70 may explain the presence of a positive correlation between the serum levels of Hsp70 and the disease progression and activity in RA (Najafizadeh et al. 2015). However, these observations are not consistent with previous pre-clinical reports that had shown that artificial immunization with highly conserved bacterial and/or murine Hsp70 peptides could be regarded as a potential treatment target for RA via induction of the antigen-specific Treg cells (van Herwijnen et al. 2012; Tukaj and Kaminski 2019). Lack of stimulatory effects of Hsp70 on the expansion of Treg in our study can be simply explained by the differences between culture conditions and the animal models. Moreover, induction of the antigen (Hsp70)-specific Treg in animal models of arthritis strictly depends on the immunological niche or the use of bacterial/murine-derived peptides. This discrepancy can be also explained by changes in the autoimmune response to Hsp70 caused by the active immunization procedure, ranging from a pro-inflammatory phenotype to a more tolerogenic functional phenotype, as it has been already described in the context of RA therapy by using an Hsp40-derived dnaJP1 peptide (Koffeman et al. 2009). Mucosal induction of the immune tolerance to dnaJP1 has led to a qualitative change from a pro-inflammatory phenotype to a tolerogenic one and to a clinical efficacy in RA patients (Koffeman et al. 2009). Moreover, the immunosuppressive activity of the extracellular autologous Hsp70 needs to be further investigated, especially in the context of its stimulatory effects on the interleukin 6 (IL-6) secretion in vitro. IL-6 is significantly increased in the sera of RA patients and is positively correlated with the disease’s activity and progression (Tukaj et al. 2010). On one hand, IL-6 produced by professional antigen presenting cells in response to different antigen stimuli can modulate the Th1/Th2 balance towards Th2 (Dienz and Rincon 2009). On the other hand, IL-6 promotes differentiation of pro-arthritis Th17 cells, blocks generation of the Treg cells, and triggers systemic inflammatory processes in RA (Schinnerling et al. 2017). Therefore, we hypothesize that the dual role of Hsp70 on Th1/Th2 and Th17/Treg T helper cell polarization is IL-6-dependent. In addition, stimulatory effects of Hsp70 on IL-6 secretion, a potent activator of the humoral (auto)immune response, might explain the presence of autoantibodies to self-Hsp70; these autoantibodies are known to be elevated in RA patients (Mantej et al. 2019). In fact, autoantibodies to Hsp70 may regulate the immune response, as evidenced by the presence of a negative correlation between the serum levels of anti-Hsp70 autoantibodies and the pro-inflammatory TNF-α in RA patients (Mantej et al. 2019). This phenomenon is also partly consistent with previous observations in regard to the other class of molecular chaperones, such as Hsp60. Naturally occurring or acquired antibodies to *M. tuberculosis* Hsp60, as well as humanized anti-Hsp60 mAb, protected against arthritis induction in an experimental model (Ulmansky et al. 2002, 2015). In addition, humanized anti-Hsp60 mAb induced secretion of the anti-inflammatory IL-10 and suppressed secretion of IFN-γ and IL-6 in the human naïve PBMC cultures (Ulmansky et al. 2015).

Taken together, this study aimed to identify the key pieces of evidence on human Hsp70 involvement in T helper cell polarization based on in vitro observations using PBMCs from healthy individuals. Although the obtained results are promising, they are nevertheless preliminary and require further investigation to validate the model we propose.
